# Exploring Perception of Indians about Plain Packaging of Tobacco Products: A Mixed Method Research

**DOI:** 10.3389/fpubh.2013.00035

**Published:** 2013-09-23

**Authors:** Monika Arora, Abha Tewari, Nathan Grills, Gaurang P. Nazar, Juhi Sonrexa, Vinay K. Gupta, Rob Moodie, K. S. Reddy

**Affiliations:** ^1^Public Health Foundation of India, Delhi, India; ^2^Health Related Information Dissemination Amongst Youth, Delhi, India; ^3^Nossal Institute for Global Health, University of Melbourne, Melbourne, VIC, Australia; ^4^Melbourne School of Population Health, Melbourne, VIC, Australia

**Keywords:** tobacco, packaging, low middle income countries, public health, health policy

## Abstract

This study assessed perceptions and support among the Indian populace about plain packaging for all tobacco products. Twelve focus group discussions (*n* = 124), stakeholder analysis with 24 officials and an opinion poll with 346 participants were conducted between December 2011 and May 2012, Delhi. Plain packages for tobacco products were favored by majority of participants (69%) and key stakeholders (92%). The majority of participants perceived that plain packaging would reduce the appeal and promotional value of the tobacco pack (>80%), prevent initiation of tobacco use among children and youth (>60%), motivate tobacco users to quit (>80%), increase notice ability, and effectiveness of pictorial health warnings on tobacco packs (>90%), reduce tobacco usage (75% of key stakeholders). Majority of participants favored light gray color for plain packaging. This study provides key evidence to advocate with Indian Government and other countries in South Asia region to introduce plain packaging legislation for all tobacco products.

## Introduction

With increasing restrictions on tobacco advertising and promotion globally, internal documents from the tobacco industry suggest that tobacco packs are valued by the industry as a means to promote their products (1). Tobacco companies utilize misleading brand imagery such as brand descriptors (light, mild, ultra-light) and pack colors (lighter shades to signify milder product and darker shades to signify stronger product) which has the potential to distract attention from the health warnings imprinted on tobacco packs (2). The use of color, fonts, images, and trademarks on tobacco packs is associated with the identity and personality of the user; these are therefore known as “badge products” (3). Repeated display of tobacco packets in social situations among both past-users and non-users is known to promote tobacco consumption norms within a social context (4). At point of sale, tobacco packs are designed to create attractive displays and promote tobacco use among youth. Research highlights that in developed countries tobacco companies experiment with producing more colorful packs, designed to stimulate curiosity among potential users (5).

To counter such industry tactics, plain packaging has been proposed under the Framework Convention on Tobacco Control (FCTC) (6). Recently, Australia became the first country to legislate a ban on the use of colors, corporate logos, trademarks, and misleading descriptors on tobacco packages (5). Manufacturers would still be required to print required health warnings and other legally mandated information such as toxic constituents, tax seals, or pack contents together with the brand name in a mandated size, font, and location (7).

Following Australia’s world-first legislation, a collaborative taskforce which included key tobacco control advocates and researchers in Australia and India, was convened to explore the feasibility of introducing plain packaging in India. It is important to expand the body of evidence relating to plain packaging in India to counter opposition from the tobacco industry and to support the Government of India in developing this process. The Cigarettes and Other Tobacco Products (Prohibition of Advertisement and Regulation of Trade and Commerce, Production, Supply, and Distribution) Act (COTPA), the tobacco control legislation in India, came into force in India since the year 2004. Key provisions of COTPA include:

Section 4 – Prohibition of smoking in public places.
Section 5 – Prohibition of tobacco advertisement, promotion and sponsorship.
Section 6 – (a) Prohibition of sale of tobacco products to and by minors; (b) Prohibition on sale of tobacco products within 100 yards of any educational institution.
Section 7 – Mandatory specified pictorial health warnings on the packaging on all tobacco products.

Cigarettes and Other Tobacco Products Act also provides for specifications related to the implementation of these laws and penalties to be imposed in case of violations. Specific details regarding COTPA are published elsewhere (8). Section 5 of COTPA pertains to prohibition of any form of direct or indirect advertising and promotion of tobacco products, in line with Article 13 of WHO FCTC (8, 9). The only exceptions to this provision include in-pack, on-pack, and point-of-sale promotion (sparing the regulations related to dimensions of point of sale advertisement boards recently upheld by the H’ble Supreme Court of India in January 2013 (10), which are still not covered under the ambit of this legislation. The tobacco industry being aware of this loop hole in the legislation employs attractive tobacco packs to advertise and promote their tobacco products as evidenced from studies conducted in developed countries.

Section 7 of COTPA, in line with Article 11 of WHO FCTC, require mandatory pictorial health warnings on all tobacco packs in India (8, 9). The guidelines require the warnings to consist of two parts (a) a graphic warning (b) accompanying text warning. The warning should occupy 40% of the principal display area of the tobacco pack; should contain specified text warning “Smoking Kills” or “Smoking Causes Cancer” on smoking forms and “Tobacco Kills” or “Tobacco Causes Cancer” on smokeless forms of tobacco (with text warning being in a language in which the brand name is mentioned); should contain specified graphic warnings, which are required to be rotated every 12 months; should not be obscured, masked, altered, or detracted from specifications provided (8). Studies conducted in developed countries have shown that attractive packs and misleading imagery used on tobacco packs distract attention from health warnings on tobacco packs.

This study aimed to assess perceptions among the Indian populace about the effectiveness of plain packaging for all tobacco products and to gauge the level of public support for plain packaging.

## Materials and Methods

### Study design and setting

This cross-sectional study was conducted from December 2011 to May 2012 in New Delhi. The study involved focus group discussions (FGDs), stakeholder analysis, and an opinion poll. FGDs and the opinion poll participants were recruited through purposive sampling to ensure equal representation by gender and socio-economic status (SES). For the FGDs, SES of adult participants was determined using location of participants’ residence (11), while among children and adolescents, those attending the government schools were considered to be representative of low SES and private school students were considered to be representative of high SES (12, 13). For the opinion poll, the 2011 revision of Kuppuswamy’s scale was used to determine SES of participants (14).

**Figure 1:**
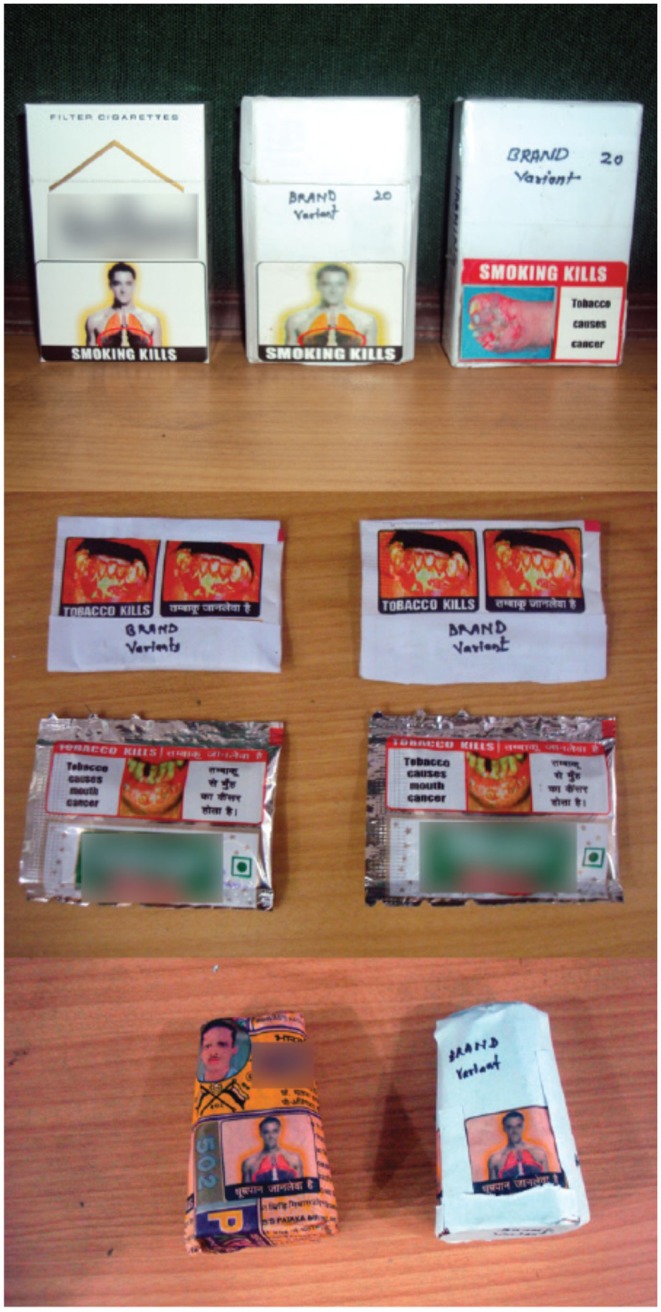
**Dummy plain packs of tobacco products**.

Informed consent was obtained from the participants and in case of minors, additional informed consent was sought from the parents. Ethics approval was obtained from the institutional ethics committee at the Public Health Foundation of India (PHFI). Dummy plain packs of tobacco products (cigarettes, *bidis*, chewing tobacco) with existing Australian warnings and plain packs with existing Indian warnings were displayed to elicit responses (Figure 1).

### Study participants and data collection

#### Focus group discussions

Eight FGDs were conducted with adult males and females (*n* = 82). Four FGDs were conducted with adult males (*n* = 44) and four with adult females (*n* = 38) between the age range of 19–64 years. Four FGDs were conducted with adolescents (*n* = 42), which included two FGDs with boys (*n* = 19) and two FGDs with girls (*n* = 23) between 12 and 17 years. The participants included both, tobacco users and non-users belonging to different socio-economic groups. Each FGD was comprised of 10–12 participants. The participants were recruited from resident welfare associations and communities. Each FGD was conducted in Hindi or English by a trained moderator assisted by a note-taker and lasted for about 45 min. The moderator conducted the FGD with the help of FGD guide (File S1 in Supplementary Material). Participants were asked to share their views and perceptions on “how important is the packaging and labeling of tobacco products,” “ how plain packaging of tobacco products affects the appeal of the tobacco products,” and so on, FGDs were audio-taped and records transcribed, translated to English and compared with the notes.

#### Stakeholder analysis

A total of 24 interviews (14 males and 10 females) were conducted with representatives of the Ministry of Health and Family Welfare (MoHFW); Department of Customs and Excise; World Health Organization; public health experts in tobacco control, cancer prevention, and behavioral sciences; community-based/non-government organizations; experts in trade and industry laws and senior faculty members from educational institutions. Interviews (15 min) were conducted by trained research staff in English using an interviewer administered questionnaire (File S2 in Supplementary Material).

### Opinion poll

The opinion poll was conducted with 346 adult participants using an interviewer administered questionnaire either in English (File S3 in Supplementary Material) or Hindi. This questionnaire was originally developed with the inputs from Australia India Task Force on plain packaging and pilot tested before the actual data collection. An equal proportion of participants were purposively selected (quota sampling) (15) in each of the following categories: male never tobacco users, male smokers, male smokeless tobacco users, male dual users (smokers and smokeless tobacco users), female never tobacco users, female smokers, female smokeless tobacco users, female dual users.

### Data analysis

Qualitative data was coded and analyzed using NVIVO 9. Thematic analysis was used in this study (16). In quantitative component, descriptive analysis and chi-square tests were used to test the differences in perceptions around plain packaging and its effectiveness between various tobacco socio-demographic groups using SPSS version 17.0.

## Results

### Focus group discussions

Responses from adults’ and adolescents’ were quite similar so their perceptions were collated together and emergent themes are presented below.

#### Knowledge of existing tobacco products

Participants were aware of tobacco products, brand names, colors, and pictorial warnings on tobacco products. They recalled specific brand names for smoked and smokeless forms of tobacco products. They could also describe colors and design and associate them with particular tobacco brands.

Nearly all participants were aware of warnings on tobacco packs and could easily describe the pictures of earlier warnings (scorpion) and the current warning (damaged lungs). A male participant from a low SES community said, “*It has an ulcerated mouth picture over it, which means that if you use the product, your mouth will also become like that; teeth are also shown to be dirty and stained.*”

#### Tobacco packs/packaging attract people

Participants generally agreed that colorful packaging lures people from all socio-economic backgrounds and age groups, especially the young, to buy tobacco products. In FGDs with adult males, the participants felt that people buy tobacco by looking at the color or appearance of packets and if packs were not attractive, people would be less interested in buying tobacco. All FGDs discussed how teenagers/youth are usually attracted to colorful packaging: “*First time when I saw it (the cigarette pack), I thought the pack contained some candies, it looked beautiful and attractive.*” said an adolescent girl. An adult male said: “*It is colorful so it attracts like [Brand Name] and so do other packets which come in different colors. Name, brand did not matter for me but I started because the packet was very attractive. Like [Brand Name], [Brand Name] or [Brand Name] used to come in brown color which looked good. We started like this only.*”

The consensus among the participants was that tobacco companies use packaging tactics to increase sales “*Tobacco companies use tactics to make packets colorful to increase their sale and production*” (a female FGD participant). The tobacco packs were perceived as a status symbol and there was an aspiration attached to their use according to FGD participants: “*The richer people have to show off their wealth, so they buy more attractive looking and expensive packs*” and “*If I am walking with an expensive cigarette packet, it will create a certain status around me*” (Males from high SES). A female student (high SES) said, “*If you bring in plain packs, those elite women who just show off the brand and style, will stop using tobacco*.” The adolescent FGDs also discussed that less educated and poorer children are more likely to be attracted to tobacco packages.

#### Awareness about plain packaging of tobacco products

Few people were aware of plain packaging prior to being recruited for the FGD. Participants shared, “*We have always seen colored, attractive products and never seen plain packs*” (Group’s view).

#### Role of plain packaging in tobacco control

When the participants were shown the dummy plain packs, their impression was that plain packaging will heavily reduce the appeal of tobacco packs especially among the youth and children. Most participants believed that young children will find these packs less appealing and so help prevent initiation and experimentation: “*I think it will make a difference as taking out a cigarette from a smart packet gives a certain style statement to the smoker or these youngsters.*” Participants thought that a plain packaging policy could contribute to reducing prevalence of tobacco use but were unsure of the effect on current tobacco users.

Female participants expressed that consumers would not buy tobacco products with less attractive packaging, for example: “*On seeing this packet they will think this (plain pack) is local and not good*” (Group’s view). Participants generally agreed that plain packages would make warnings more prominent, more “*in your face*” as a male high SES student put it. Some participants expressed concern that if only cigarettes were included in the policy, then people might shift to other tobacco products with attractive packing.

#### Suggestions provided by the participants

Participants suggested that larger, colored pictorial warnings should replace the brand name, as it would help deter people from using tobacco products. Colors like black, white, brown (dull shade), and grey were suggested to be least appealing. Light grey was most favored: “*The background should be light gray and the picture should be brighter so that the pictorial warning gets more emphasis*” (a male adolescent from low SES).

### Opinion poll

Overall, 346 participants were surveyed (52.9% male; 43.7% low SES, 39.9% mid SES, and 16.4% high SES). Most participants were literate (84.1%). About 24.3% of participants were unemployed, 21.1% unskilled workers, and 19.4% professionals. The median age was 31 years (IQR = 25–40 years). About 44.8% had never used any tobacco products, 55.4% were ever tobacco users (current + past tobacco users), and 51.2% were current tobacco users (35% smokers, 36.7% smokeless tobacco users, and 28.3% dual users) (Table 1).

**Table 1:** **Demographic profile of participants and tobacco use prevalence (*N* = 346)**.

	***N***	%
**GENDER**		
Male	183	52.9
Female	163	47.1
**AGE**		
18–33	200	57.8
34–49	110	31.9
50 and above	35	10.1
**EDUCATION**		
Illiterate	55	15.9
Primary school certificate	53	15.3
Middle school certificate	36	10.4
High school certificate	60	17.3
PG diploma	26	7.5
Graduate or post-graduate	95	27.5
Advanced professional degree (e.g., PhD etc.)	21	6.1
**OCCUPATION**		
Unemployed	84	24.3
Unskilled worker (laborer)	73	21.1
Semi-skilled worker	40	11.6
Skilled worker	23	6.7
Clerical, shop owner, farmer	24	7.0
Semi-professional	34	9.9
Professional	67	19.4
**SOCIO-ECONOMIC STATUS**		
Low	149	43.7
Middle	136	39.9
Upper	56	16.4
**TOBACCO USE PREVALENCE**		
Current tobacco user	177	51.2
Past tobacco user	14	4.0
Never user	155	44.8
**CURRENT USERS (*N* ***=*** 177)**		
Smoke	62	35.0
Smokeless	65	36.7
Both	50	28.3

#### Perceptions about pictorial health warnings and brand imagery

Overall 28.1% of participants usually noticed pictorial health warnings first when they looked at a tobacco pack while 53% noticed branding (brand name, color, and design). Younger people (18–33 years) noticed branding more than the pictorial warnings. More low SES respondents noticed pictorial warnings (32%) than those from high SES, who noticed branding more (*data not shown*).

#### Perception about tobacco packs

About 76% of participants felt tobacco packs were attractive, 83.2% reported that colors, designs, gloss, and large fonts of brand name on the tobacco pack distract a consumer from the pictorial health warning and 86.7 and 83.8% of the participants felt that the tobacco industry uses attractive packaging to lure adults and adolescents respectively into using their products. More participants from the upper SES group compared with lower SES reported that current tobacco packs caused distraction from pictorial health warnings (100 vs. 75%, *p* < 0.001) and that the tobacco industry uses attractive packaging to lure children and adolescents into using their products (96.4 vs. 79.7%, *p* < 0.05). No significant differences were observed between responses by gender, or between different age groups. Compared with current tobacco users (79%), more never-users (90.3%) reported that attractive packaging is being used to lure children and adolescents (*p* < 0.05) (Table 2).

**Table 2:** **Perceptions about current tobacco packs on overall look and attractiveness, by demographic profile and tobacco use status of the respondents**.

	Attractive, *n* (%)	Distract from pictorial warnings, *n* (%)	Lure adults, *n* (%)	Lure children and adolescents, *n* (%)
Overall	264 (76.3)	288 (83.2)	300 (86.7)	290 (83.8)
**GENDER**				
Male	136 (74.7)	147 (81.7)	159 (87.4)	155 (85.2)
Female	128 (78.5)	141 (86.5)	141 (86.5)	135 (82.2)
*p-*Value	0.405	0.223	0.813	0.553
**AGE**				
18–33	152 (76.0)	175 (87.5)	179 (89.5)	172 (86.0)
34–49	85 (77.3)	86 (79.6)	95 (86.4)	94 (85.5)
50 and above	26 (76.5)	26 (76.5)	26 (76.5)	24 (70.6)
*p-*Value	0.969	0.092	0.104	0.068
**SOCIO-ECONOMIC STATUS**				
Low	107 (72.3)	111 (75.0)	128 (86.5)	118 (79.7)
Middle	104 (76.5)	118 (87.4)	114 (83.8)	115 (84.6)
Upper	49 (87.5)	55 (100.0)	53 (94.6)	54 (96.4)
*p-*Value	0.074	<0.001	0.131	0.013
**TOBACCO USE PREVALENCE**				
Current tobacco user	133 (75.6)	139 (79.4)	148 (84.1)	139 (79.0)
Past tobacco user	10 (71.4)	13 (92.9)	11 (78.6)	11 (78.6)
Never user	121 (78.1)	136 (88.3)	141 (91.0)	140 (90.3)
*p-*Value	0.780	0.059	0.114	0.016
**CURRENT USERS**				
Smoke	50 (80.6)	51 (82.3)	55 (88.7)	52 (83.9)
Smokeless	44 (67.7)	50 (76.9)	53 (81.5)	52 (80.0)
Both	39 (79.6)	38 (79.2)	40 (81.6)	35 (71.4)
*p-*Value	0.176	0.757	0.466	0.270

#### Brand value among participants

About 57% of participants felt that various tobacco brands are different in how prestigious they are and 49% of participants perceived that various tobacco brands are different in how attractive they are to consumers (*data not shown*).

#### Perceptions about effectiveness of plain packaging

About 69% of participants strongly approved of the plain packaging proposal. There were no significant differences in this finding across socio-demographic groups. Only 5.5% participants somewhat or strongly disapproved of this proposal. The majority of participants agreed that plain packaging would reduce the attractiveness of tobacco products among both users and non-users (81.8 and 83.2%, respectively); it could motivate tobacco users to quit (83.2%) and could also make pictorial warnings more effective (91.6%). Participants across different demographic profiles and users of different tobacco products (smoked, smokeless, and both) had similar responses. More current tobacco users than never-users reported that plain packaging can reduce the attractiveness of tobacco products among both users and non-users (*p* < 0.05) (Table 3).

**Table 3:** **Perceptions about plain packaging on its effectiveness, by demographic profile and tobacco use status of the respondents**.

	Can reduce the attractiveness of tobacco product		Can motivate tobacco users to quit	Can make the pictorial warnings effective
	Among users, *n* (%)	Among non-users, *n* (%)	*n* (%)	*n* (%)
Overall	283 (81.8)	288 (83.2)	288 (83.2)	317 (91.6)
**GENDER**				
Male	149 (84.2)	148 (81.8)	153 (84.5)	171 (94.0)
Female	134 (83.8)	140 (85.9)	135 (83.3)	146 (90.1)
*p-*Value	0.914	0.301	0.763	0.187
**AGE**				
18–33	163 (83.2)	167 (83.9)	171 (85.9)	184 (92.5)
34–49	89 (84.0)	89 (81.7)	86 (79.6)	102 (93.6)
50 and above	30 (88.2)	31 (88.6)	30 (85.7)	30 (85.7)
*p-*Value	0.759	0.622	0.341	0.312
**SOCIO-ECONOMIC STATUS**				
Low	119 (81.0)	117 (79.6)	120 (81.1)	133 (89.9)
Middle	115 (87.1)	115 (84.6)	117 (86.7)	127 (94.1)
Upper	45 (83.3)	52 (92.9)	46 (83.6)	54 (96.4)
*p-*Value	0.376	0.069	0.446	0.197
**TOBACCO USE PREVALENCE**				
Current tobacco user	154 (88.0)	154 (87.5)	145 (82.4)	160 (90.4)
Past tobacco user	9 (64.3)	9 (64.3)	12 (85.7)	13 (92.9)
Never user	120 (81.1)	125 (81.2)	131 (85.6)	144 (94.1)
*p-*Value	0.029	0.040	0.716	0.453
**CURRENT USERS**				
Smoke	54 (87.1)	53 (86.9)	51 (83.6)	56 (90.3)
Smokeless	57 (89.1)	60 (92.3)	58 (89.2)	60 (92.3)
Both	43 (87.8)	41 (82.0)	36 (72.0)	44 (88.0)
*p-*Value	0.942	0.249	0.053	0.739

About 48% of participants felt that plain packaging with the Australian pictorial warnings would more effectively discourage non-users from initiating tobacco use and 60.7% perceived that they would encourage users to quit as compared with current tobacco packs (6.6 and 5.2% respectively) and plain packs with a current Indian warning (44.8 and 32.7% respectively).

### Stakeholder analysis

#### Packaging and pictorial health warnings

About 96% of respondents were aware of Indian packaging and labeling requirements for tobacco products and agreed that tobacco products are attractively packaged by the industry. Over 75% of the participants felt that color, design, and graphics on a tobacco pack are aspects that particularly render the pack attractive.

### Plain packaging

Most respondents (83.3%) were aware of the plain packaging proposal and three quarters believed that it will reduce tobacco usage. Some of the reasons stated by the stakeholders were (a) with loss of attractiveness of packets, youth might be less likely to initiate tobacco use (b) it would help control direct or indirect/surrogate advertisements. Nearly all stakeholders considered plain packaging of tobacco products relevant to the Indian context.

**Figure 2:**
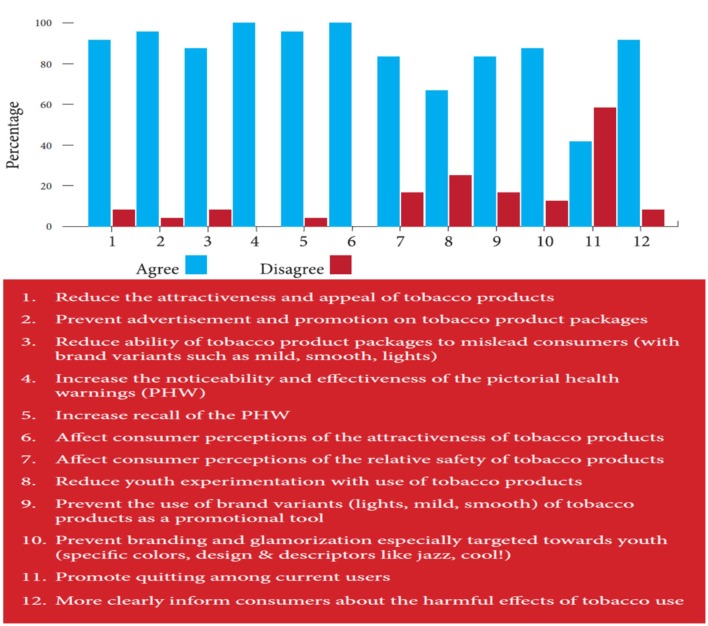
**Perceptions of key stakeholders about the impact of plain packaging**.

About 75% of stakeholders said it was possible to adopt plain packaging in India as it was about the right to good health. Most respondents supported the impact of plain packaging across the 11 fields (Figure 2) with the only exception being its effect on quitting.

### Implementation of plain packaging

#### Barriers and key facilitators

Lack of political will, tobacco industry opposition, and issues with pan-India implementation were identified as the three most common challenges for implementation of plain packaging in India. Others included:

- laying groundwork by building evidence
- trademark issues
- weak compliance with FCTC
- diversity of tobacco products in India
- unregulated market for bidi and smokeless forms
- sale of loose tobacco
- difficulty with product differentiation among various brands
- large numbers of manufacturers
- social acceptability

Multi-sectoral partnerships were suggested as key to plain packaging implementation and the three most important stakeholders identified were: (a) policymakers, the most important being from the MoHFW, (b) law enforcers, and (c) civil society groups and NGOs. Other identified partners were: Ministries of Commerce and Industry, Law and Justice, Agriculture and Co-operation and Rural Development.

#### Law and plain packaging (cigarettes and other tobacco products act, copyright and trade laws)

Stakeholders mentioned that as per the provisions of COTPA, pictorial warnings are already in place and they could be strengthened by amending Section 7 of COTPA to introduce plain packaging. Some stakeholders suggested amendment to Section 5 of COTPA, which would involve deleting the proviso (exception extended to the tobacco industry) of Section 5(2), which allows advertisements on tobacco packs. About 33% of stakeholders thought that copyright and trade laws might adversely affect implementation of plain packaging in India, but 58% of the stakeholders thought it would not.

## Discussion

This study explored the promotional value of tobacco packaging, attitudes toward plain packaging, and resultant challenges and opportunities for such a policy as perceived by Indian populace and key stakeholders. As observed in earlier studies outside India, the consensus among participants in this study was that tobacco companies intentionally make their product packs attractive (17). A particular finding was that the “style” factor associated with handling an expensive brand made the user feel affluent. This is important as it might encourage low SES groups to spend more on tobacco in order to mimic those belonging to high SES groups. Similar to a recent opinion poll on plain packaging conducted in UK (18), our study demonstrated a 70% support including key stakeholders in favor of plain packaging.

Despite the differences observed among current and never tobacco users, majority of (over 80%) participants consistently reported that plain packaging would reduce the attractiveness appeal and promotional value of tobacco products and their packaging among both users and non-users of tobacco. Attractiveness of tobacco packaging is an attribute that research from developed countries shows is used by tobacco companies to promote their products (1). Among current users, attractive packaging provides a reinforcement mechanism for continued use of tobacco. An earlier experimental study conducted in Australia suggested that smoking cigarettes from plain packs was perceived to be less satisfying by smokers compared with smoking cigarettes from packs with full branding and other imagery (3). Our findings support this observation suggesting that plain packaging is expected to remove the positive reinforcement associated with attractive tobacco packs which would render smoking less satisfying among current users, thereby supporting cessation efforts. Among non-users, attractive pack imagery would imply drawing their attention toward the tobacco packs to encourage experimentation and initiation. The Australian experimental study also suggested that smokers of cigarettes from plain packs were perceived to be less attractive, popular, stylish, and mature compared with smokers from packs with full branding and other imagery (3). Our findings complement this observation suggesting that plain tobacco packs, by removing the “style statement” associated with attractive tobacco packs, would prevent experimentation and initiation of tobacco among non-users of tobacco.

Most study participants suggested that plain packaging would likely prevent experimentation and initiation of tobacco use among youth. Studies on plain packaging in developed countries show that plain packaging is associated with increased negative perceptions and feelings about the pack and smoking, avoidant behavior such as hiding or covering the pack, smoking cessation behaviors such as decreased smoking, skipping of smoking episodes, and thinking about quitting (19). Perceptions of the majority of our participants about the effectiveness of plain packaging in quitting are therefore in accordance with these previous studies. Still, some participants in the FGDs and stakeholder analysis were skeptical as to whether plain packaging would encourage quitting amongst current tobacco users.

Moreover, it was also suggested that plain packaging would increase the impact and notice ability of the pictorial warnings (which in turn would lead to increased knowledge about the health effects of tobacco use). Earlier, a study conducted in the UK found that plain packaging increased visual attention toward the health warning (20). The “dual effect” arising from an increasingly plainer, unattractive pack, and increased attention toward a large and effective pictorial health warning appears to be the major reason for the perceived effectiveness of plain packs as demonstrated in developed countries.

As observed in Australia (21), strong industry opposition is anticipated in India and is perceived as a major challenge. A number of study participants feared that political will – as demonstrated by the Australian Government – might be lacking in India (22). When contemplating plain packaging, policymakers, and decision makers in countries such as India need to consider the additional challenges highlighted and their impacts. For example, in India, study participants detailed how the plain packaging law would need to apply to all forms of tobacco products including smokeless tobacco products, the prevalence of which is higher in the Indian context. Failing this, there would be the possibility of a substitution of unregulated products.

The stakeholders outlined that India has the advantage of having comprehensive tobacco control legislation in the form of COTPA (6). The existing provisions (Section 5 and 7) of COTPA could be amended to incorporate plain packaging. India also benefits from a dedicated National Tobacco Control Program (corroborating the support from Ministry of Health and Family Welfare) (23), a supportive print and electronic media and a strong civil society alliance – the Advocacy Forum for Tobacco Control – a network of civil society organizations (24). Stakeholders in this study stated that these partnerships and resources need to be mobilized to counter challenges such as anticipated industry resistance.

This study revealed that dull colors (e.g., light grey) were most favored. Interestingly, in Australia, drab dark brown was the favored color for plain packaging and light grey was thought to look to smart and silvery (25). However, further large-scale studies representative of urban and rural participants are required to ascertain the most effective color for plain packaging in the Indian setting.

The study is limited in size and geographical representation; notably, rural areas were not included. However, the triangulation of methods and variety of data sources minimizes this limitation somewhat. Reassuringly, results from across different sources and from different methods were largely consistent. This study is an excellent starting point, but the limitations indicate that further evidence is required in order to build the case for plain packaging policy in India.

## Conflict of Interest Statement

The authors declare that the research was conducted in the absence of any commercial or financial relationships that could be construed as a potential conflict of interest.

## Supplementary Material

The Supplementary Material for this article can be found online at http://www.frontiersin.org/Public_Health_Education_and_Promotion/10.3389/fpubh.2013.00035/abstract
