# Supplementary material for: Exploring Perception of Indians about Plain Packaging of Tobacco Products: A Mixed Method Research
Source: Front Public Health. 2013 Sep 23;1:35. doi: 10.3389/fpubh.2013.00035 (PMC3859976; doi:10.3389/fpubh.2013.00035)
Supplement: Supplementary file 1 [file 59613_Arora_Presentation1.PDF]

Supplement file1

Questions for the Focus Group Discussions:

1. Have you ever seen any tobacco product (cigarette, gutkha and bidi packaging? Please describe your impressions on seeing a tobacco pack. (*Probe: packet design, colour scheme, brand names*). According to you, how important is the packaging and labelling of any tobacco product? Do you think the current packaging and labelling of tobacco products promote tobacco products?

2. Can you recognize which tobacco product is mild/strong or less harmful from package appearance? (*Probe for any misleading messages/misbelieve/false perception)*
   - Did the packaging of the product play a role in your decision to use or not to use tobacco product?

3. Have you ever seen the pictorial health warnings on any tobacco product? If yes, please describe the warnings that you have seen (spontaneous recall). If no, aid the recall by describing the text warning first (*Probe: packet design, pictures, colour scheme, message, brand names)*.

4. In your opinion what are the reasons for displaying these pictorial warnings on tobacco products? *(Probe: provision of law, government initiative to create awareness among people about tobacco menace, protecting youth and adults from tobacco use, strength of pictorial warnings vs. identification of brand name)*

5. Have you seen/ heard about the plain packaging on tobacco products? How and Where? Please share your experiences? *(Probe: packet design, colour scheme, brand names)*.

6. (Please show the dummy pack & discuss). Do you think Plain packaging of tobacco products are helpful and effective to
   - Enhance pictorial warnings which inform tobacco users about health risks of tobacco use.
   - Affect the appeal of the tobacco product in terms of the appeal of the pack.
   - Adults and children will find these packs less appealing, less palatable and less satisfying.

- Prevent branding and glamorization especially targeted towards youth
- Prevent children / youth/adults tobacco use
- Helps in reducing tobacco use rates
- Helps in increasing consumer knowledge about health risks of tobacco use
- Support tobacco users to quit.

7. (Please show the dummy pack and discuss)If you compare both the packs which one is more helpful and effective in discouraging from initiating/experimenting tobacco use?*(Probe : gender, SES, literacy, youth vs. adults)*

8. In your opinion which is more helpful and supportive in reducing and quitting tobacco use among users.*(Probe : gender, SES, literacy, youth vs. adults)*

9. Any additional information that can be put up in these plain packaging. Please suggest.

Supplement File:2

Questionnaire for Stakeholders

Name:____

Designation:____

Department/organization:__________________________________

State:_____________________

Email:_____________________ **Phone:**___________

Place:_____________________ **Date:**___/___/______

Questions

1. **Are you familiar with the packaging and labeling requirements of tobacco products in India?**

   a. Yes ☐
   b. No ☐

2. **How do you rate the clarity of the current pictorial health warnings on tobacco products in informing you about the health hazards of tobacco use?**
   a. Very clear ☐
   b. Clear ☐
   c. Somewhat clear ☐
   d. Not Clear ☐
   e. Confusing ☐

3. **Do you think tobacco products are attractively packaged?**
   a. Yes ☐
   b. No ☐

   If answered No, skip to Q6.

4. **What elements of the tobacco product pack make them attractive?**
   a. Colour ☐
   b. Shape ☐
   c. Design/Graphics ☐
   d. Text ☐
   e. Size ☐
   f. Others (Please specify): ____

5. **Such color and design elements of a tobacco product package are carefully chosen by the tobacco industry to attract and/or promote tobacco products among: (Mark all that apply)**

   a. Children and adolescents ☐
   b. Women ☐
   c. Men ☐
   d. Aged ☐
   e. Others (Please specify): ____

6. **Do you think the current pictorial health warnings can be made more effective?**

   a. Yes ☐
   b. No ☐
   c. Don't know ☐

   If yes, what do you suggest (chose options from below, you can √ multiple options):

   a. Health effects of tobacco clearly visible in pictures ☐
   b. Multiple health effects to be informed by rotation every year ☐
   c. Neutral/unappealing background color for all tobacco products ☐
   d. Uniform pack design for all brands of tobacco products ☐
   e. Stronger health warning text ☐
   f. All of the above ☐
   g. Others (please specify)______________________

7. **Are you aware of plain packaging for tobacco products?**

   a. Yes ☐
   b. No ☐

   If no, show dummy packs

8. **Do you think plain packaging of tobacco products can decrease tobacco usage?**

   a. Yes ☐
   b. No ☐
   c. Don't know ☐

9. **Do you think plain packaging is relevant to Indian Context?**

   a. Very relevant ☐
   b. Relevant ☐
   c. Somewhat relevant ☐
   d. Irrelevant ☐
   e. Don't know ☐

   If answered a-d, why? ____

10. **Will it be possible to adopt plain packaging in India?**

    a. Certainly ☐
    b. May be ☐
    c. Can't say ☐
    d. May be not ☐
    e. Not at all ☐

    If answered a, b, d or e, state why?

    ____

11. **List top three challenges or barriers, in your opinion, in implementing plain packaging in India?**

    a. ____
    b. ____
    c. ____

12. **Who according to you are the three most important/influential partners in implementing this policy?**

    a. ____
    b. ____

c. ____

13. **How might this policy be supported by COTPA?**

____

____

14. **What according to you are the weaknesses of COTPA in implementing this policy?**

____

____

15. **Do you think COTPA should be amended/will need amendment to introduce plain packaging?**

a. Yes ☐
b. No ☐
c. Not sure ☐

If yes, then please suggest some amendments which are relevant as per your view

………………………………………………………………………………………………
………………………………………………………………………………………………
………………………………………………………………………………………………
………………………

16. **Do you think copyright and trade laws will adversely affect implementing of plain packaging in India?**

a. Yes ☐
b. No ☐

If yes, how?

____

17. **Do you think introducing plain packaging will:** (*Please √ against applicable*)

**Sl no.**	**Reason for introducing plain packaging**	**Strongly Agree**	**Agree**	**Can't Say**	**Disagree**	**Strongly Disagree**
a.	Reduce the attractiveness and appeal					

	of tobacco products					
b.	Prevent advertisement and promotion on tobacco products packages					
c.	Reduce ability of tobacco products packages to mislead consumers (with brand variants such as mild, smooth, lights)					
d.	Increase the noticeability and effectiveness of the pictorial health warnings					
e.	Increase recall of the pictorial health warnings					
f.	Affect consumer perceptions of the attractiveness of the tobacco products					
g.	Affect consumer perceptions of the relative safety of the tobacco products					
h.	Reduce youth experimentation with use of tobacco products					
i.	Prevent the use of brand variants (lights, mild, smooth) of tobacco products as a promotional tool					
j.	Prevent branding and glamorization especially targeted towards youth (specific colors, design and descriptors like jazz, cool)					
k.	Promote quitting among current users					
l.	More clearly inform consumers about the harmful effects of tobacco use					

Supplement File3

Opinion Poll Questionnaire

- ***Tick only one answer unless otherwise specified***

Details of the Interviewee

Gender Male ☐ Female ☐

Age ☐☐ (in years)

Education (*Tick highest qualification only*)

a. Advanced professional degree (Eg. PhD etc.) ☐
b. Graduate or Post-graduate ☐
c. PG Diploma ☐
d. High School Certificate ☐
e. Middle School Certificate ☐
f. Primary School Certificate ☐
g. Illiterate ☐

Current Occupation

a. Professional (e.g. doctors, nurses, lawyers, engineers etc.) ☐
b. Semi-Professional (technicians, assistants etc.) ☐
c. Clerical, shop owner, farmer ☐
d. Skilled worker (e.g. carpenter, plumber etc. **with** formal training or certificate) ☐
e. Semi-skilled worker(e.g. carpenter, plumber etc. **without** any formal training or certificate) ☐
f. Unskilled worker (Labourer) ☐
g. Unemployed ☐

Monthly family income from all sources

a. ≥ 29,766 ☐
b. Rs. 14,883-29,765 ☐
c. Rs. 11,162-14,882 ☐
d. Rs. 7442-11,161 ☐
e. Rs. 4465-7441 ☐
f. Rs. 1503-4464 ☐
g. ≤ 1502 ☐

1. Please select one of the following options:
   a. I currently use tobacco products ☐
   b. I have used tobacco product in the past but I have quit now ☐ --- **Quit since ___ months**
   c. I have never used any tobacco products ☐ --- **Go to Q3**

2. Which product do you use now or used in the past? (***Tick all that apply***)
   a. Cigarette ☐
   b. Bidi ☐
   c. Chewing tobacco (gutka, zarda, khaini etc) ☐
   d. Others (Please specify) ☐
      ____

3. Based on what you know or believe, are there any illnesses or diseases caused by tobacco use?
   a. Yes ☐----**Go to Q4**
   b. No ☐----**Go to Q5**
   c. Refused ☐----**Go to Q5**
   d. Don't know ☐----**Go to Q5**

4. Which diseases or illnesses are caused by tobacco use (smoking or use of smokeless tobacco)? (***Tick all that apply***) (***ONLY PROBE: Any others? Others?; ASK SEPARATELY FOR SMOKING AND SMOKELESS FORMS; DO NOT PROBE FOR INDIVIDUAL DISEASES***)

**Disease/Illness**	**Response**	
	**Smoking forms**	**Smokeless forms**
1. Respiratory Diseases (Bronchitis, Asthma, emphysema coughing etc.)		
2. Cardiovascular Diseases (Heart disease, gangrene, hypertension, stroke etc.)		
3. Various types of cancers (Lung, Oral, Breast, Bladder, etc.)		
4. Diabetes		
5. Eye problems (e.g. blindness, etc)		
6. Reproductive problems (e.g. impotence, infertility etc.)		
7. Child related problems (e.g. sudden death, low birth weight babies, infections etc.)		
8. Infections (e.g. Pneumonia, tuberculosis, flu/cold, etc.)		
9. Kidney problems		
10. Mental illnesses (e.g. depression, anxiety etc.)		
11. Hygiene problems (e.g. bad breath, stained teeth, etc.)		

12. Others		

5. When you look at a tobacco pack, what do you usually notice first?
   a. Warning labels ☐
   b. Other aspects such as branding (including brand name, colour, design) ☐
   c. Something else on the pack (Please specify) ☐
   ____
   d. Refused ☐
   e. Don't know ☐

Show current pictures on tobacco packs

6. Do these pictorial warnings effectively inform you about the hazards of tobacco use?
   a. Surely yes ☐
   b. Maybe yes ☐
   c. Maybe no ☐
   d. Surely no ☐
   e. Refused ☐
   f. Don't know ☐

7. To what extent do you think the pictorial health warnings currently on tobacco packs motivate ***users to quit***?
   a. Not at all ☐
   b. Somewhat ☐
   c. Greatly ☐
   d. Refused ☐
   e. Don't know ☐

8. To what extent do you think the pictorial health warnings currently on tobacco packs motivate ***non-users* to stay quit**?
   a. Not at all ☐
   b. Somewhat ☐
   c. Greatly ☐
   d. Refused ☐
   e. Don't know ☐

9. How often do you think that seeing a tobacco pack would lead you to think that tobacco can't be as harmful as the health experts say?

a. Never ☐
b. Rarely ☐
c. Sometimes ☐
d. Often ☐
e. Very often ☐
f. Refused ☐
g. Don't know ☐

10. Do you think tobacco product packs are attractive?
    a. Surely yes ☐
    b. Maybe yes ☐
    c. Maybe no ☐
    d. Surely no ☐
    e. Refused ☐
    f. Don't know ☐

11. Do you think that colours, designs, gloss and large fonts of brand name on the tobacco pack distract a consumer from the pictorial health warning?
    a. Surely yes ☐
    b. Maybe yes ☐
    c. Maybe no ☐
    d. Surely no ☐
    e. Refused ☐
    f. Don't know ☐

12. Do you think that the tobacco industry uses attractive packaging to lure ***adults*** into using their products?
    a. Surely yes ☐
    b. Maybe yes ☐
    c. Maybe no ☐
    d. Surely no ☐
    e. Refused ☐
    f. Don't know ☐

13. Do you think that the tobacco industry uses attractive packaging to lure ***children and adolescents*** into using their products?
    a. Surely yes ☐
    b. Maybe yes ☐
    c. Maybe no ☐
    d. Surely no ☐
    e. Refused ☐
    ☐

f. Don’t know

14. In your opinion, how different are tobacco brands ……… (***Tick the appropriate box for all three***)

	Not at all different	Somewhat different	Very different	Refused	Don’t know
a. In how prestigious they are					
b. In how harmful they are					
c. In how attractive they are to adolescents and other consumers					

15. Tobacco companies often use tobacco packaging for promotional purposes. Because of this, some health authorities in other countries have proposed that tobacco should be sold in plain packaging (Show dummy packs). This means that all packs would look the same with logos and colours removed. The brand name in plain text and pictorial health warnings would still remain on the packs. Overall, do you approve or disapprove of this plain packaging proposal?

    a. Strongly approve ☐
    b. Somewhat approve ☐
    c. Somewhat disapprove ☐
    d. Strongly disapprove ☐
    e. Refused ☐
    f. Don’t know ☐

16. Do you think plain packaging (show dummy packs) of tobacco products can reduce the attractiveness of tobacco products? (***Ask for both, users as well as non-users***)

	Among users	Among non-users
a. Surely yes		
b. Maybe yes		
c. Maybe no		
d. Surely no		
e. Refused		
f. Don’t know		

17. Do you think plain packaging (show dummy packs) of tobacco products can motivate tobacco users to quit tobacco use?
    a. Surely yes ☐
    b. Maybe yes ☐
    c. Maybe no ☐
    d. Surely no ☐
    e. Refused ☐
    f. Don't know ☐

18. Do you think plain packaging (show dummy packs) of tobacco products makes the pictorial warnings more effective?
    a. Surely yes ☐
    b. Maybe yes ☐
    c. Maybe no ☐
    d. Surely no ☐
    e. Refused ☐
    f. Don't know ☐

19. Which of these tobacco packs do you think is more effective in discouraging non-users from initiating tobacco use?
    a. Pack 1: current packaging (current pictorial warning) ☐
    b. Pack 2: plain packs (with current pictorial warnings) ☐
    c. Pack 3: plain pack (with an effective Australian pictorial warning) ☐

20. Which of these tobacco packs do you think is more effective in encouraging users to quit?
    a. Pack 1: current packaging (current pictorial warning) ☐
    b. Pack 2: plain packs (with current pictorial warnings) ☐
    c. Pack 3: plain pack (with an effective Australian pictorial warning) ☐

21. In your opinion, plain packs of which colour will be more effective in decreasing the appeal and attractiveness of tobacco packaging?
    a. Brown ☐
    b. Green ☐
    c. Grey ☐
    d. White ☐
    e. Black ☐
    f. Others (Please specify) ☐ ____________________________
    g. Refused ☐
    h. Don't know ☐
